# Hydrocodone Rescheduling and Opioid Prescribing Disparities in Breast Cancer Patients

**DOI:** 10.3390/cancers17132146

**Published:** 2025-06-25

**Authors:** Chan Shen, Mohammad Ikram, Shouhao Zhou, Roger Klein, Douglas Leslie, James Douglas Thornton

**Affiliations:** 1 Department of Surgery, College of Medicine, The Pennsylvania State University, Hershey, PA 17033, USA; mikram2@pennstatehealth.psu.edu; 2 Department of Public Health Sciences, College of Medicine, The Pennsylvania State University, Hershey, PA 17033, USA; szhou1@pennstatehealth.psu.edu (S.Z.); dleslie@pennstatehealth.psu.edu (D.L.); 3 Penn State Cancer Institute, Hershey, PA 17033, USA; 4 Department of Economics, Rutgers University, New Brunswick, NJ 08901, USA; rklein@economics.rutgers.edu; 5 Department of Pharmaceutical Health Outcomes and Policy, University of Houston College of Pharmacy, Houston, TX 77204, USA; jdthornt@central.uh.edu; 6 Prescription Drug Misuse Education and Research (PREMIER) Center, University of Houston College of Pharmacy, Houston, TX 77204, USA

**Keywords:** hydrocodone rescheduling, breast cancer, opioids, race–ethnicity, SEER, Medicare, Medicaid, pain management

## Abstract

**Background**: Pain is a prevalent issue among breast cancer patients and survivors, with a significant proportion receiving hydrocodone for pain management. However, the rescheduling of hydrocodone from Schedule III to Schedule II by the U.S. Drug Enforcement Administration (DEA) in October 2014 raised concerns about potential barriers to opioid access for cancer patients, particularly among vulnerable populations such as dually eligible Medicare–Medicaid beneficiaries and racial/ethnic minorities. **Methods**: We conducted a retrospective cohort study using Surveillance, Epidemiology, and End Results (SEER)-Medicare linked data including 52,306 early-stage breast cancer patients from 2011 to 2019. We employed multivariable logistic regression models with model specification tests to stratify the subgroups and evaluate the differential effects of the policy change by Medicaid dual eligibility and race–ethnicity, while adjusting for other patient demographics, clinical characteristics, and cancer treatments. **Results**: The rescheduling of hydrocodone was associated with significantly different effects on prescription opioid use across subgroups, with the most pronounced reduction in hydrocodone prescription observed among dual-eligible racial/ethnic minority patients (adjusted odds ratio [AOR] = 0.57; 95% confidence interval [CI]: 0.44–0.74; *p* < 0.001). Non-dual-eligible patients experienced a smaller reduction in hydrocodone use (AOR = 0.84; 95% CI: 0.78–0.90; *p* < 0.001). Concurrently, non-hydrocodone opioid use significantly increased among non-dual-eligible non-Hispanic White patients (AOR = 1.29; 95% CI: 1.19–1.40; *p* < 0.001), suggesting a substitution effect, while smaller non-significant increases were observed among other subgroups. **Conclusions**: Hydrocodone rescheduling led to the greatest reduction in hydrocodone use among dual-eligible racial–ethnic minority patients. The corresponding increase in non-hydrocodone opioid use was limited to non-dual-eligible non-Hispanic White patients. These findings highlight the need for opioid policies that balance misuse prevention with equitable access to pain relief, particularly among underserved populations.

## Simple Summary

This study evaluated the differential impacts of the 2014 hydrocodone rescheduling policy on opioid prescribing patterns among early-stage breast cancer patients, using SEER-Medicare data from 2011 to 2019. Among 52,306 patients, we stratified analyses according to Medicaid dual eligibility and racial/ethnic status. Hydrocodone rescheduling was associated with a significant reduction in hydrocodone use across all groups, with the largest decrease observed among dual-eligible racial/ethnic minority patients (AOR = 0.57). Concurrently, non-hydrocodone opioid use significantly increased only among non-dual-eligible non-Hispanic White patients (AOR = 1.29), suggesting a substitution effect that was not evident in other groups. These findings raise concerns about access to effective pain management following regulatory changes. Our results underscore the need for opioid policies that both prevent misuse and ensure equitable access to pain management, particularly for socioeconomically disadvantaged and minority cancer populations.

## 1. Introduction

Pain is a prevalent and significant concern for breast cancer patients and survivors. Approximately 42% of breast cancer survivors and 47% of patients undergoing breast cancer treatment experience pain [1,2]. Studies indicate that 13% of these patients report severe pain, while 39% experience moderate pain [3]. Prescription opioids are widely regarded as the gold standard for pain management in breast cancer patients, with hydrocodone being the most commonly prescribed opioid in the United States for over a decade [4,5].

Effective pain management is crucial for improving the health-related quality of life in cancer patients [6]. However, substantial disparities in access to pain management persist, particularly among underserved and underprivileged populations [7,8,9,10,11,12]. Research has shown that cancer patients covered by Medicaid and those from racial and ethnic minority groups report higher pain scores and greater unmet pain management needs, which not only diminish the quality of life but also impact treatment adherence and exacerbate disparities in cancer outcomes [9,11,12]. Additionally, breast cancer prevalence is higher among dual-eligible beneficiaries (Medicare and Medicaid) than among Medicare-only patients [13], and racial and ethnic minorities also have higher cancer prevalence and worse outcomes [13,14,15]. A beneficiary is considered dual-eligible if they are enrolled in Medicare (Part A and/or Part B) and receive any level of Medicaid assistance—either full Medicaid benefits or partial assistance through a Medicare Savings Program [16].

Meanwhile, the misuse of controlled substances, including prescription opioids, remains a critical public health issue associated with severe consequences such as dependency, psychotic disorders, and death [17,18]. In response, numerous policy efforts have been implemented to curb opioid misuse. One significant policy change was the rescheduling of hydrocodone from Schedule III to Schedule II in October 2014 by the U.S. Drug Enforcement Administration (DEA) [19]. This rescheduling prohibited refills of hydrocodone, requiring a new hard-copy or electronic prescription for each fill, and restricted call-in or faxed prescriptions except in emergency situations [19,20]. While most stakeholders in the cancer care community recognize the necessity of preventing opioid misuse, concerns have been raised that such policy changes may introduce barriers to pain management for cancer patients who rely on opioids, potentially compromising their quality of life [21].

It has been shown that the rescheduling of hydrocodone from Schedule III to Schedule II in 2014 led to a documented 20–30% nationwide decline in hydrocodone dispensing [22]. However, prior opioid control policies—such as Prescription Drug Monitoring Program (PDMP) mandates and state-level prescribing limits—have not impacted all populations equally [23]. Evidence suggests that racial/ethnic minority patients and dual-eligible Medicare–Medicaid beneficiaries often experience disproportionately larger reductions in opioid dispensing, raising concerns about equity in access to pain management [24]. Against this policy backdrop, we hypothesized that hydrocodone rescheduling would (i) reduce hydrocodone use overall; (ii) lead to the greatest reductions among dual-eligible and racial/ethnic minority patients; and (iii) be accompanied by compensatory increases in non-hydrocodone opioid use, primarily among non-dual-eligible, non-Hispanic White patients. Although previous studies have examined the impact of this policy change on pain management in cancer patients [25,26], none have specifically assessed its differential effects on breast cancer patients according to dual eligibility and racial/ethnic status. Evaluating policy interventions is essential to understanding their effectiveness in preventing opioid misuse while ensuring adequate pain management and informing future policy efforts [27]. Given the significant socioeconomic and racial–ethnic disparities in pain management, it is critical to consider how policy changes may differentially impact these vulnerable populations.

In this study, we utilize a large, nationwide, population-based dataset to evaluate the differential impacts of hydrocodone rescheduling on the use of both hydrocodone and non-hydrocodone prescription opioids in breast cancer patients, stratified according to dual eligibility and racial/ethnic status. We focused on a national cohort of older adults in the United States with early-stage breast cancer—a population uniquely suited for examining opioid policy effects. Early-stage breast cancer patients typically undergo surgery, with nearly 75% receiving an opioid prescription at discharge and 10–20% developing persistent opioid use [28]. Survivorship pain, including persistent post-surgical pain, affects up to 60% of patients [29]—a higher value than in many other cancer types. Focusing on this clinically homogeneous group with both high opioid exposure risk and substantial life expectancy provides an ideal sentinel population for assessing equity in opioid policy impacts. SEER-Medicare was selected as our data source for its unique combination of high-quality cancer registry data and comprehensive healthcare claims [16,30]. The SEER program is a nationally recognized source for population-based cancer surveillance, collecting detailed information on newly diagnosed cancer cases—including diagnosis date, cancer site, stage, tumor characteristics, and initial treatment—from multiple states across the United States. These records are securely linked to Medicare claims, which provide longitudinal data on inpatient, outpatient, and prescription drug utilization. In addition, SEER-Medicare provides detailed information on race/ethnicity and dual eligibility. Together, SEER-Medicare enables the robust investigation of cancer care, health services use, and treatment outcomes in a large, diverse population of older adults.

## 2. Methods

### 2.1. Data Source

The data source utilized in this study was the Surveillance, Epidemiology, and End Results (SEER) registry linked with Medicare claims for Medicare beneficiaries in the United States, who were aged 65 years and older from 2011 to 2019. The SEER registry, which is supported by the National Cancer Institute (NCI), is an authoritative repository for population-based cancer research, representing over 35% of the population in the United States [31]. By establishing a linkage between SEER data and Medicare claims, the dataset is enhanced with supplementary information regarding healthcare utilization both preceding and following cancer diagnosis for Medicare beneficiaries, encompassing Parts A, B, and D. Since their inception in 2007, Medicare Part D claims have offered detailed records pertaining to pharmaceutical prescriptions, which include prescription opioids, as well as various other medications. The combined dataset provides an extensive array of data concerning patient age, sex, race, ethnicity, Medicare insurance benefit categories, neighborhood socioeconomic status, tumor type, staging, a large range of cancer treatments received, pharmaceutical pain management, and survival outcomes.

### 2.2. Study Cohort

The study cohort comprised females aged 66 years and above diagnosed with early-stage incident breast cancer, with early-stage breast cancer being defined as AJCC 6th Edition Stage I breast cancer treated with primary surgery. Patients whose diagnoses were established solely through autopsy reports were excluded from this analysis. We identified patients diagnosed between 1 January 2011 and 31 December 2018, with follow-up extending for one year until the end of 2019, a timeframe that captures the 2014 policy change on hydrocodone rescheduling while excluding the COVID-19 pandemic period beginning in 2020. We assessed comorbidities based on healthcare utilizations recorded in the year preceding the cancer diagnosis, and we conducted follow-up for one year to examine their pain management pharmacotherapy after diagnosis. Consequently, we required patients to maintain continuous enrollment in Medicare Parts A, B, and D, without health maintenance organization (HMO) coverage, from one year prior to diagnosis until the conclusion of the first year post-diagnosis to ensure comprehensive records for the identification of pre-existing comorbidities, medications pertinent to pain management, and cancer treatment throughout the study timeframe. We restricted the cohort to patients aged 66 years and older to ensure at least 12 months of continuous Medicare enrollment prior to cancer diagnosis, which is necessary for reliably capturing comorbidities and other baseline characteristics using claims data. Although Medicare eligibility typically begins at age 65, a minimum age of 66 allows for one full year of look-back data. We excluded patients enrolled in Medicare Advantage (Health Maintenance Organization—HMO) plans because claims for these beneficiaries are not consistently available in the SEER-Medicare dataset, limiting the completeness and comparability of utilization data. HMOs also represent a different care delivery model from traditional fee-for-service Medicare, which may further affect prescribing patterns.

### 2.3. Patient Characteristics

The key patient characteristics that are the focus of this study are Medicaid dual eligibility (yes vs. no) and race–ethnicity (non-Hispanic White vs. racial–ethnic minority including non-Hispanic Black, Hispanic/Latino, and others). In addition to these characteristics, we also considered patient age at breast cancer diagnosis, Charlson comorbidity score [32], the presence of clinically diagnosed depression, and neighborhood socioeconomic status, including census tract-level income quartile, the percentage of residents without a high school degree, and the percentage below the poverty line, as these may have considerable influence on pain management. Moreover, we incorporated the various cancer treatments administered (radiation, chemotherapy, immunotherapy, and hormonal therapy) into our analysis, as these interventions can significantly affect the strategies employed for pain management. The Charlson comorbidity score and the cancer treatments were based on ICD-9, ICD-10, and CPT/HCPCS codes captured in insurance claims data. The covariates included in the analyses were selected based on an extensive review of the literature, which identified factors known to influence opioid prescribing and pain management, limited to those that were available and reliably measured in the SEER-Medicare dataset [33,34,35,36].

### 2.4. Prescription Opioids

We analyzed two categories of prescription opioids—hydrocodone and non-hydrocodone opioids. The utilization of these pharmacologic treatments was determined based on the generic drug names listed in patients’ Medicare Part D pharmaceutical claims. Non-hydrocodone opioids included codeine, dihydrocodeine, fentanyl, hydromorphone, buprenorphine, methadone, levorphanol, meperidine, morphine, opium, oxycodone, oxymorphone, tapentadol, and tramadol.

### 2.5. Statistical Analysis

We performed univariate analyses to describe the sample characteristics, presenting the frequencies and percentages for all variables included in the study. We used chi-square tests to examine subgroup differences according to hydrocodone and non-hydrocodone opioid use. To assess the differential effects of policy changes and the overall temporal trends across dual eligibility and racial/ethnic subgroups, we conducted multivariable logistic regression analyses that controlled for patient characteristics, including age, race–ethnicity, Medicaid dual eligibility, depression, Charlson comorbidity score, and cancer treatments. The effect of the policy change was evaluated based on a binary indicator that distinguished the periods before and after the rescheduling of hydrocodone in October 2014, while the temporal trend was assessed using a continuous variable, representing time in months. We conducted large-sample Wald chi-square tests for model specification [37]. Based on these model specification tests, the sample was stratified into three groups to examine the differential impacts on hydrocodone use—non-dual-eligible, dual-eligible non-Hispanic White, and dual-eligible racial–ethnic minority. For non-hydrocodone opioid use, the sample was categorized into three groups—dual-eligible, non-dual-eligible non-Hispanic White, and non-dual-eligible racial–ethnic minority. Details of the model specification test results will be discussed in the results section. To illustrate the temporal trends, we plotted monthly time trends from 2011 to 2019 in the percentage of patients using hydrocodone and non-hydrocodone prescription opioids by the subgroups.

As a sensitivity analysis, we conducted segmented time series logistic regression models that included terms for the pre-policy linear time trend, an indicator variable to capture any immediate level change at the time of policy implementation, and an interaction term to estimate changes in the post-policy trend. This approach allowed us to quantify the annual change in the odds of hydrocodone use before and after the policy, as well as any immediate shifts in prescribing behavior associated with the policy introduction.

To further explore the implications of opioid prescribing patterns, we conducted a supplementary analysis examining long-term opioid use, which was defined as ≥90 days of hydrocodone or non-hydrocodone prescriptions within the first year post-diagnosis. This threshold is commonly used in the literature to characterize chronic or persistent opioid use [38,39,40].

We define a substitution effect as any statistically significant increase in non-hydrocodone opioid use that coincides with a decline in hydrocodone prescribing following the DEA’s 2014 rescheduling, which is consistent with the frameworks used in prior evaluations of opioid policy impacts [41]. This concept reflects potential shifts in prescribing behavior from one class of opioid to another in response to regulatory restrictions. We use the term “barriers to access” to refer to systemic or structural factors that may limit certain populations’ ability to obtain clinically appropriate opioid medications [42,43]. In this context, we consider policy-driven constraints—such as more restrictive Medicare Part D formularies, prior authorization requirements, or geographic disparities in pharmacy availability—as barriers. This usage aligns with prior definitions of access in health services research, which conceptualize access as both the availability of services and the ability of patients to use them.

All statistical analyses were conducted using SAS version 9.4 (SAS Institute, Cary, NC, USA). For the multivariable logistic regression models, we reported adjusted odds ratios (AORs) with 95% confidence intervals (CIs) and corresponding *p*-values. All statistical tests were two-sided, with *p*-values less than 0.05 being considered statistically significant. The Penn State Institutional Review Board approved this retrospective study based on large observational data.

## 3. Results

The study included a total of 52,306 breast cancer patients, with a nearly even distribution across age groups—24.3% were aged 66–69, 29.2% were aged 70–74, 21.5% were aged 75–79, and 25.0% were aged 80 or older. The majority of the sample was non-Hispanic White (83.1%), while 6.1% were non-Hispanic Black, 5.7% were Hispanic/Latino, and 5.1% identified as another race–ethnicity. A total of 18.0% of patients were dually eligible for Medicare and Medicaid. Regarding pain management, 45.8% of patients received hydrocodone, while 47.6% were prescribed non-hydrocodone opioids. Cancer treatment modalities included radiation therapy (58.2%), chemotherapy (28.5%), immunotherapy (14.1%), and hormonal therapy (89.6%). Comorbidity burden varied, with 42.3% having zero Charlson comorbidities, 28.1% having one, 16.9% having two, and 12.7% having three or more; a total of 21.4% had a diagnosis of depression. Table 1 presents the detailed sample descriptives. The results of the subgroup comparisons for hydrocodone and non-hydrocodone opioid use are provided in Supplementary Table S1.

To present the results of the multivariable logistic regression analyses examining the policy impact on hydrocodone use, we first describe the model specification test results that guided the stratification of the study sample. The model specification test identified a significant difference (*p* = 0.011) between dual-eligible and non-dual-eligible patients, indicating that these groups responded differently to the rescheduling of hydrocodone. Among dual-eligible patients, further stratification was warranted, as the model specification test detected significant differences between non-Hispanic White and racial–ethnic minority patients (*p* = 0.003), underscoring substantial racial–ethnic differences even within the dual-eligible population. In contrast, among non-dual eligible patients, the model specification test showed no significant differences according to race–ethnicity (*p* = 0.269), suggesting that race–ethnicity did not play a significant role in hydrocodone use patterns within this group.

Multivariable logistic regression analyses, stratified according to race–ethnicity groups informed by the above model specification tests, revealed significant reductions in hydrocodone use following the policy change across all subgroups. However, the magnitude of these effects varied substantially. Among dual-eligible non-Hispanic White patients, the adjusted odds of hydrocodone use significantly decreased post-policy change (AOR = 0.75; 95% CI: 0.60–0.94; *p* = 0.013) (Table 2), with a reduction of 12 absolute percentage points. The most pronounced reduction was seen among dual-eligible racial–ethnic minority patients, for whom the policy change was associated with a 43% reduction in the adjusted odds of hydrocodone use (AOR = 0.57; 95% CI: 0.44–0.74; *p* < 0.001) and a reduction of 17 absolute percentage points. Among non-dual-eligible patients, hydrocodone use also declined post-policy change, though the magnitude of the impact was much less pronounced (AOR = 0.84; 95% CI: 0.78–0.90; *p* < 0.001) with a reduction of 11 absolute percentage points. In all three subgroups, we observed an overall continuous decline over time in the use of hydrocodone with AORs ranging from 0.90 to 0.95. Such results are in line with our hypotheses (i) and (ii).

For the analysis of non-hydrocodone use, the model specification test again identified significant differences according to dual eligibility status (*p*-value = 0.025). However, within the dual-eligible group, there were no significant differences according to race–ethnicity (*p*-value = 0.350); in contrast, significant race–ethnicity differences (*p*-value = 0.044) were identified among non-dual-eligible patients. The multivariable logistic regression analysis, stratified according to the above subgroups, revealed mixed effects following the policy change, with statistically significant increases observed only among non-dual-eligible non-Hispanic White patients. In this subgroup, the odds of non-hydrocodone opioid use significantly increased post-policy change (AOR = 1.29; 95% CI: 1.19–1.40; *p* < 0.001) (Table 3) with a reduction of 9 absolute percentage points. Among non-dual-eligible patients, there was a moderate increase in non-hydrocodone opioid use after the policy change (AOR = 1.22; 95% CI: 0.97–1.53) with a reduction of 8 absolute percentage points, although this result did not reach statistical significance (*p* = 0.087). For dual-eligible patients, the policy change was associated with the smallest non-significant increase in non-hydrocodone opioid use (AOR = 1.12; 95% CI: 0.95–1.32; *p* = 0.178) with a reduction of 5 absolute percentage points. No significant time trend effects were detected in all three subgroups with AORs ranging from 1.00 to 1.03. Such results are in line with our hypothesis (iii).

In addition, we found that receipt of chemotherapy, radiation therapy, and a higher Charlson comorbidity score were each associated with modestly increased odds of both hydrocodone and non-hydrocodone opioid use, consistent with findings from prior research [44,45,46]. These associations likely reflect greater symptom burden and pain management needs among patients with more intensive treatment or comorbid conditions. Detailed results are presented in Supplementary Table S2.

The sensitivity analysis confirmed the robustness of our primary findings. Hydrocodone use was already declining prior to the policy. The 2014 rescheduling was associated with a large, immediate drop in prescribing, but it did not significantly alter the existing downward trajectory—the decline continued at approximately the same rate post-policy. For non-hydrocodone opioid use, we observed a modest upward trend before the policy, a temporary spike coinciding with the policy’s implementation, and a subsequent tapering—consistent with a short-term substitution effect. The detailed results are provided in Supplementary Table S3.

**Figure 1:**
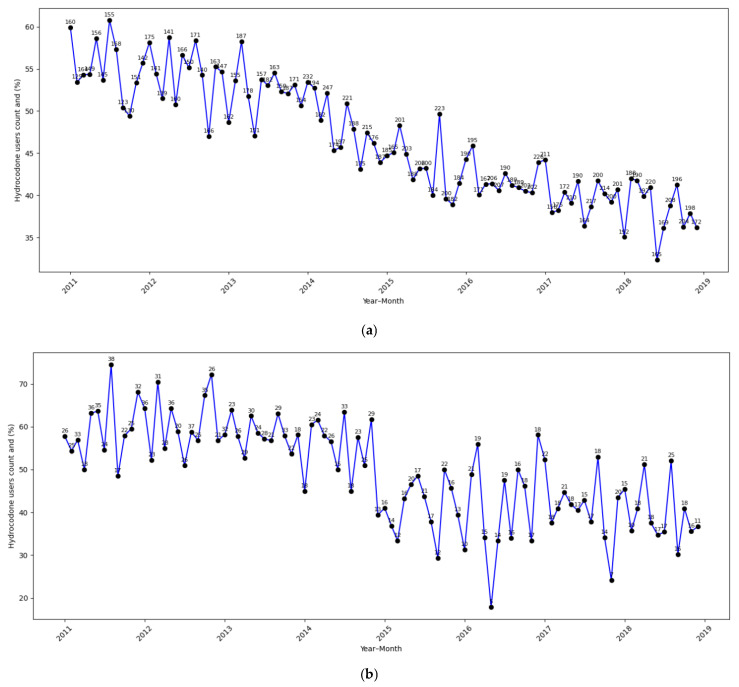

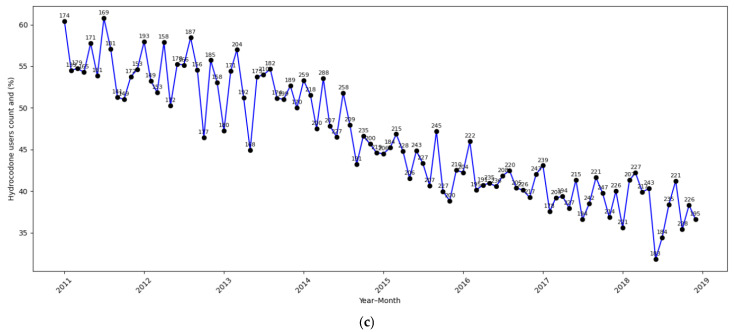
(**a**): Dual-eligible non-Hispanic White. (**b**): Dual-eligible racial–ethnic minority. (**c**): Non-dual-eligible.

Supplementary Table S4 provides the results from the supplementary analysis on long-term opioid use. In contrast to our main findings for hydrocodone use, this supplementary analysis showed that the policy change was not significantly associated with long-term hydrocodone use in any subgroup. Among dual-eligible non-Hispanic White patients (AOR = 0.81; 95% CI: 0.58–1.14; *p* = 0.228) and dual-eligible racial–ethnic minority patients (AOR = 0.88; 95% CI: 0.54–1.44; *p* = 0.609), post-policy reductions were statistically non-significant. Among non-dual eligible patients, long-term hydrocodone use was stable (AOR = 1.03; 95% CI: 0.83–1.29; *p* = 0.783), though a statistically significant downward trend over time was observed (AOR = 0.89 per 12 months; *p* < 0.001), suggesting a broader secular decline. Similarly, no statistically significant changes were observed in long-term non-hydrocodone opioid use post-policy change across any subgroup. The strongest signal—a borderline decline—was seen among non-dual-eligible non-Hispanic White patients (AOR = 0.65; 95% CI: 0.42–1.01; *p* = 0.056). Across all groups, time-related trend effects were non-significant. These results suggest that while short-term prescribing behavior was responsive to the policy change, long-term opioid use—potentially representing patients with sustained pain needs—was less affected. This may indicate that providers maintained continuity in pain management for patients requiring prolonged opioid therapy, reflecting appropriate clinical judgment. Figure 1a–c illustrate the monthly time trends in the percentage of patients using hydrocodone, providing a visual representation consistent with the logistic regression results. A consistent post-policy decline was observed across all subgroups, with the steepest reduction being seen among dual-eligible racial–ethnic minority patients (Figure 1b), aligning with the strongest policy impact detected in the regression analysis. Dual-eligible non-Hispanic White patients (Figure 1a) and non-dual-eligible patients (Figure 1c) also exhibited decreasing trends, though the drop in hydrocodone use was less pronounced than the dual-eligible racial–ethnic minority subgroup.

**Figure 2:**
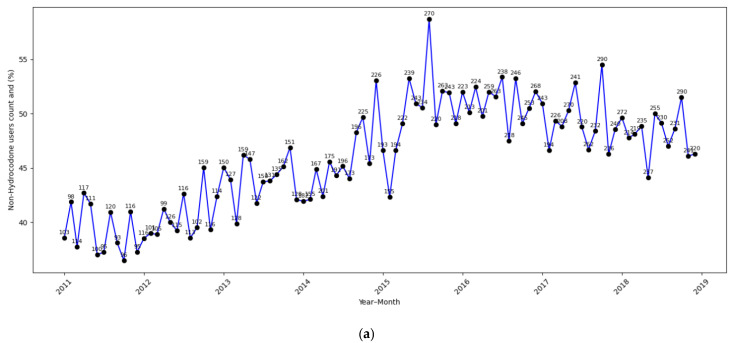

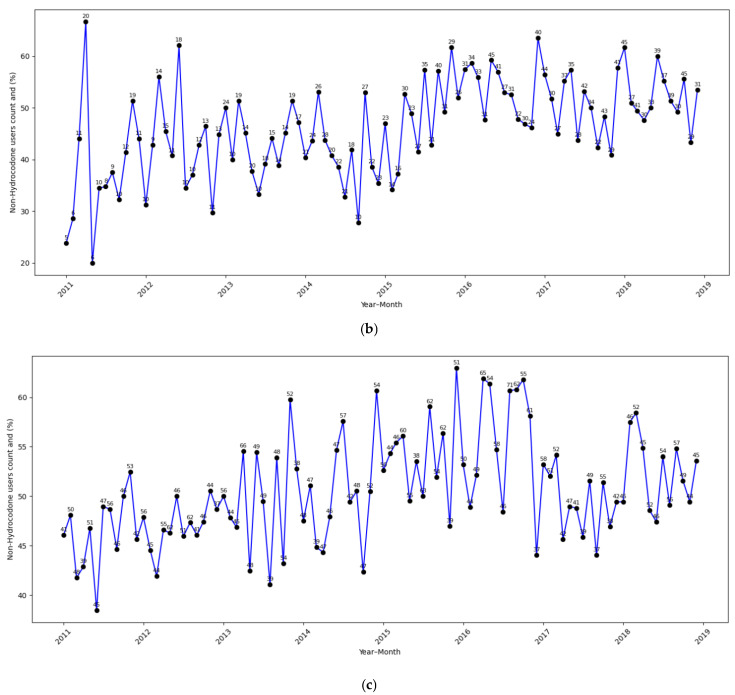
(**a**): Non-dual eligible non-Hispanic White. (**b**): Non-dual-eligible racial–ethnic minority. (**c**): Dual-eligible.

Figure 2a–c display the time-related trends in non-hydrocodone opioid use across different subgroups. In alignment with the regression findings, non-hydrocodone opioid use significantly increased among non-dual-eligible, non-Hispanic White patients (Figure 2a) after the policy change. In contrast, the trends showed less of an increase for non-dual-eligible racial–ethnic minority patients (Figure 2b) and dual-eligible patients (Figure 2c), consistent with our findings from the logistic regression.

## 4. Discussion

To the best of our knowledge, this is the first study to specifically examine the differential impacts of hydrocodone rescheduling from a Schedule III to a Schedule II controlled substance on the use of both hydrocodone and non-hydrocodone prescription opioids among breast cancer patients according to dual eligibility and racial–ethnic groups. Our findings indicate significant differences in response to this policy change within these subgroups. The reduction in the odds of hydrocodone use was most pronounced among dual-eligible racial–ethnic minority patients (43%), suggesting that this population experienced the most substantial shift in opioid prescribing practices. In contrast, the non-dual-eligible group exhibited the smallest reduction (16%) in the odds of hydrocodone use. Notably, hydrocodone use reduction did not differ significantly according to race/ethnicity among non-dual-eligible patients. Conversely, non-hydrocodone opioid use increased primarily among non-dual-eligible non-Hispanic White patients (29%), indicating the strongest potential substitution effect following the policy change in this group. In contrast, dual-eligible patients experienced a small and non-significant increase in the use of non-hydrocodone prescription opioids, with no significant difference according to race/ethnicity in this subgroup.

These results suggest that dual eligibility had a strong differential impact on the response to hydrocodone rescheduling, with the reduction in hydrocodone use being more pronounced in dual-eligible patients, while the increase in non-hydrocodone use was more pronounced in non-dual-eligible patients. Among dual-eligible patients, racial–ethnic minorities experienced the largest decrease in hydrocodone use, while among non-dual-eligible patients, non-Hispanic White patients had the largest increase in non-hydrocodone prescriptions, potentially substituting hydrocodone for pain management purposes. The combination of a substantial decrease in hydrocodone use and a minimal increase in non-hydrocodone use among dual-eligible racial–ethnic minority patients warrants further investigation into the potential barriers to accessing prescription opioids following hydrocodone rescheduling. The substantial reduction in high-dose hydrocodone prescriptions and shorter supplies following rescheduling [47]—consistent with national Medicare trends—suggests that part of the observed decline likely reflects improvements in opioid stewardship rather than restricted access alone. However, the lack of a corresponding increase in non-hydrocodone prescribing among dual-eligible racial/ethnic minority patients indicates that these stewardship benefits may not have been equitably distributed. Several actionable strategies can be pursued. These include targeted prescriber education on equitable pain management, the integration of clinical decision support tools to prompt safe and appropriate opioid substitutions, and Medicaid formulary reforms—such as removing prior authorization requirements for guideline-concordant analgesic alternatives. To mitigate fear of addiction and stigma surrounding opioid use among racial and ethnic minority populations, strategies may include culturally competent patient education, provider training to reduce implicit bias, and the integration of behavioral health and palliative care services. Partnering with trusted community organizations and ensuring public health messaging distinguishes appropriate medical use from misuse can also help normalize opioid use for cancer pain and promote equitable pain management. These steps could help reduce administrative barriers and promote the more consistent application of opioid stewardship across diverse patient populations.

The interpretation of declines in hydrocodone use is limited by the absence of patient-reported pain severity, functional outcomes, and quality-of-life data in SEER-Medicare. As a result, we cannot definitively distinguish between improved opioid stewardship and the potential undertreatment of pain. Prior natural-experiment studies have found that reduced opioid prescribing does not necessarily lead to worse patient-reported outcomes or satisfaction [48]. However, national surveys consistently report poorer pain control among Black and Latino adults, despite lower opioid exposure [49]. This suggests that observed reductions in prescribing may not be experienced uniformly and could reflect unmet pain needs in some populations. Further research incorporating patient-centered outcomes is needed to clarify the impact of opioid policy changes on the adequacy of pain management.

While no prior studies have examined the differential impacts of hydrocodone rescheduling on opioid prescribing patterns and barriers to access, the existing literature has demonstrated disparities in opioid prescribing. Studies have shown that non-Hispanic White patients are more likely to receive opioid prescriptions compared to racial–ethnic minority patients [50,51], while dual-eligible patients face significant barriers to pain management services [52,53]. For example, one study found that White patients were prescribed opioids up to 80% more frequently than Black patients and up to 25% more frequently than Hispanic patients following opioid-related events [54]. Other studies have indicated that dual-eligible patients might face challenges such as limited provider availability, fragmented care, inadequate coverage, and socio-structural barriers [52,53,55]. Our findings, which suggest increasing barriers to prescription opioids among dual-eligible racial–ethnic minorities, are particularly concerning given prior evidence that these groups report higher rates of high-impact chronic pain. For instance, a study found that 23.9% of full-/partial-benefit dual-eligible beneficiaries reported high-impact chronic pain, compared to only 10.5% of non-dual-eligible individuals between 2018 and 2020 [56]. Similarly, other studies have found that African American and Hispanic patients report a higher pain intensity compared to non-Hispanic White patients [57].

Although SEER-Medicare lacks information on plan formularies or pharmacy-level drug availability, prior research indicates that Medicare Part D plans serving low-income and racial/ethnic minority beneficiaries tend to apply more restrictive utilization controls on opioids. In addition, pharmacies located in predominantly Black or Latino neighborhoods are significantly less likely to stock a full range of opioid medications [58,59]. These structural barriers may limit the ability of dual-eligible racial/ethnic minority patients to substitute hydrocodone with other opioids following the 2014 rescheduling. In contrast, non-Hispanic White beneficiaries—who are more likely to be enrolled in less-restrictive plans and reside in areas with better pharmacy access—may have been more readily able to substitute hydrocodone with agents like codeine or tramadol [60,61], consistent with national substitution trends. Future studies incorporating plan-level formulary information or pharmacy access indices could more directly test these hypothesized mechanisms.

Findings from our study combined with the existing literature on pain management disparities highlight the need for equitable opioid prescribing practices to prevent worsening disparities in pain management. Balanced opioid policies should aim to prevent misuse while ensuring access for patients with legitimate pain management needs, particularly among vulnerable populations. Prior research suggests that drug rescheduling may lead to short-term increases in total deaths due to some opioid users transitioning to heroin as an alternative [62]. Given this, it is especially important to monitor pain management needs and consider alternative pain management strategies for dual-eligible racial–ethnic minority patients.

Our findings align with and contextualize recent federal efforts to balance opioid stewardship with equitable access to pain management. The 2018 SUPPORT Act mandated opioid drug utilization reviews within Medicare and Medicaid and catalyzed CMS’ broader opioid strategy, including demonstration projects aimed at expanding substance use disorder treatment capacity [63]. In parallel, CMS implemented opioid safety edits in Medicare Part D, such as the 7-day supply limit for opioid-naïve patients, as well as the nationwide rollout of Drug Management Programs (DMPs) targeting high-risk beneficiaries [64]. These interventions, introduced shortly after hydrocodone rescheduling, represent a national shift toward more closely monitored opioid prescribing. Importantly, such policy changes may have amplified effects among dual-eligible and racially minoritized beneficiaries, who are disproportionately enrolled in Medicaid-linked Part D plans. Many studies have shown that state-level Medicaid policies—including coverage restrictions, prior authorization requirements, and reimbursement limits—can significantly influence opioid prescribing and access within these populations. These policy variations may help explain some of the disparities observed in our study [65,66].

This study has several limitations. As an observational study, although our analysis controlled for various demographic and clinical factors, unmeasured confounders may still influence the findings. For example, the SEER-Medicare dataset lacks detailed information on provider prescribing preferences, patient-reported pain severity, functional status, cancer recurrence, and treatment intensity, all of which are potential confounders. Unmeasured influences at both the provider and regional levels may be particularly relevant. Prior research has documented significant racial disparities in opioid prescribing at the individual provider level, as well as wide variation in hydrocodone substitution patterns across specialties following the 2014 DEA rescheduling [67,68,69]. In addition, the geographic maldistribution of pain management services—particularly in rural areas—may further limit access to alternative analgesic therapies, disproportionately affecting vulnerable populations. Future studies incorporating provider identifiers, county-level prescriber density, and measures of geographic access (e.g., distance to care) are warranted to better understand and disentangle these contextual effects. Moreover, the geographic maldistribution of pain management services—particularly in rural areas—disproportionately limits access to alternative analgesic therapies [70,71]. The incorporation of provider identifiers, county-level prescriber density, and distance-to-care measures could further disentangle these contextual effects.

Additionally, our study relied on Medicare Part D claims data, which do not capture non-prescription and illicit opioid use, such as heroin, illicit fentanyl, or diverted prescription opioids, potentially underestimating opioid consumption patterns. The SEER-Medicare dataset lacks direct information on plan formularies, prior authorization policies, or real-time pharmacy stock. However, several national studies demonstrate that coverage restrictions and drug availability vary systematically according to race/ethnicity and insurance status, offering plausible contextual explanations for the asymmetric substitution we observed [58,59,72]. Nevertheless, such interpretation needs to be considered with caution.

Further, the SEER-Medicare dataset lacks validated measures of pain severity, quality of life, and functional status; therefore, we cannot directly assess the adequacy of analgesia in this cohort. As such, any inference that certain groups may experience unmet pain needs is speculative and should be interpreted with caution. Future research could include prospective studies that evaluate the long-term impact of opioid policy changes on cancer-related pain outcomes, with attention being paid to patient-reported measures and sociodemographic factors. Such research could incorporate patient-reported outcomes to assess how changes in opioid prescribing practices affect actual pain relief and overall well-being to minimize unintended consequences, such as inadequate pain relief or increased reliance on illicit substances. Qualitative studies examining patient experiences and clinician perspectives on opioid prescribing post-policy change would provide a deeper understanding of the barriers and facilitators to effective pain management. Investigating how healthcare providers balance opioid prescribing guidelines with individual patient needs will be crucial for developing policies that optimize both safety and pain relief.

Another limitation is the generalizability of our findings; as our study population is limited to fee-for-service beneficiaries aged ≥ 66 years with continuous Medicare enrollment, the results may not generalize to younger patients, Medicare Advantage enrollees, or individuals without stable coverage. Our findings should be interpreted within the context of early-stage breast cancer. This study excluded patients with stage III or IV disease; therefore, we cannot infer the effects of hydrocodone rescheduling on patients with metastatic breast cancer or other cancer types, where pain prevalence, opioid needs, and treatment trajectories differ substantially. The post-rescheduling decline in hydrocodone use and the observed race-by-dual eligibility substitution patterns may not extend to patients with advanced or metastatic disease, where pain burden is substantially higher. Pain prevalence increases from approximately 40% in the post-curative setting to over 60% in advanced-stage breast cancer [73], and patients with metastatic disease often experience more severe symptom burden [74]. These patients also receive opioids at significantly higher rates and doses, frequently requiring longer-term pain management. Additionally, early and consistent engagement with palliative care has been shown to improve long-acting opioid prescribing and overall pain control. However, dual-eligible and racial/ethnic minority patients are less likely to receive palliative care and hospice services in a timely manner and are more likely to undergo intensive end-of-life interventions [75]. Limited access to these supportive services may have further constrained opioid substitution in patients with advanced or metastatic disease. Future studies should extend this line of inquiry to advanced cancer populations and other tumor types to assess the broader implications of opioid policy changes across the cancer care continuum. Our exclusion of Medicare Advantage (HMO) enrollees, due to incomplete claims data, may limit the generalizability of our findings to patients under HMO plans. HMO plans may have different care coordination structures and prescribing protocols, which could result in distinct opioid use patterns [76,77].

## 5. Conclusions

Nevertheless, this study provides valuable insights into the differential impact of hydrocodone rescheduling on opioid prescribing patterns among breast cancer patients stratified according to dual eligibility and racial–ethnic groups. Our findings underscore the need for careful consideration of how opioid policy changes affect different patient populations, particularly those who are socioeconomically disadvantaged or belong to racial–ethnic minority groups. By highlighting the disparities in opioid prescribing and access following regulatory changes, this study contributes to the broader discourse on equitable pain management. Moving forward, policymakers and healthcare providers should implement strategies that balance opioid misuse prevention with the necessity of ensuring adequate pain relief for all patients, particularly the most vulnerable populations.

## Figures and Tables

**Table 1:** Sample description of the study.

	Total (N = 52,306)
**Age**	
66–69	12,727 (24.3%)
70–74	15,277 (29.2%)
75–79	11,229 (21.5%)
≥80	13,073 (25.0%)
**Race/Ethnicity**	
Non-Hispanic White	43,459 (83.1%)
Non-Hispanic Black	3192 (6.1%)
Hispanic/Latino	2984 (5.7%)
Other	2671 (5.1%)
**% of Non-high school degree**	
0–<5%	5713 (10.9%)
5% to <10%	7942 (15.2%)
10% to <20%	12,690 (24.3%)
20% to 100%	25,899 (49.6%)
**% below poverty**	
0–<5%	13,817 (26.4%)
5% to <10%	11,701 (22.4%)
10% to <20%	13,875 (26.6%)
20% to 100%	12,851 (24.6%)
**Income quartile**	
First quartile	13,412 (25.6%)
Second quartile	12,795 (24.5%)
Third quartile	12,727 (24.3%)
Fourth quartile	13,372 (25.6%)
**Hydrocodone**	
Yes	23,979 (45.8%)
No	28,327 (54.2%)
**Non-Hydrocodone opioids**	
Yes	24,897 (47.6%)
No	27,409 (52.4%)
**Radiation therapy**	
Yes	30,455 (58.2%)
No	21,851 (41.8%)
**Chemotherapy**	
Yes	14,905 (28.5%)
No	37,401 (71.5%)
**Immunotherapy**	
Yes	7351 (14.1%)
No	44,955 (85.9%)
**Hormonal therapy**	
Yes	46,880 (89.6%)
No	5426 (10.4%)
**Dual Eligibility**	
Yes	9393 (18.0%)
No	42,913 (82.0%)
**Depression**	
Yes	11,200 (21.4%)
No	41,106 (78.6%)
**Charlson comorbidity**	
No	20,258 (42.3%)
1	13,431 (28.1%)
2	8110 (16.9%)
3 or more	6054 (12.7%)
**Policy Change**	
Pre-policy	20,254 (38.7%)
Post policy	32,052 (61.3%)
**Continuous month**	
N (Missing)	52,306 (0)
Mean (SD)	53.4 (26.98)
Median (Range)	56.0 (1.0, 96.0)

**Table 2:** Multivariable logistic regression results for hydrocodone use.

Hydrocodone Use				
	Variables	AOR	95% CI	*p*-Value
Dual-eligible non-Hispanic White				
Policy Change				
	Post policy change	0.75	[0.60, 0.94]	<0.013
	Before policy change (reference)			
Time Trend				
	in 12 months	0.90	[0.86, 0.95]	<0.001
Dual-eligible racial–ethnic minority				
Policy Change				
	Post policy change	0.57	[0.44, 0.74]	<0.001
	Before policy change (reference)			
Time Trend				
	in 12 months	0.95	[0.90, 1.00]	0.063
Non-dual-eligible				
Policy Change				
	Post policy change	0.84	[0.78, 0.90]	<0.001
	Before policy change (reference)			
Time Trend				
	in 12 months	0.91	[0.90, 0.93]	<0.001

Note: The regression models were controlled for age groups, income quartiles, percent below poverty level, percent below non-high school degree, radiation, chemotherapy, immunotherapy, hormonal therapy, depression, and Charlson comorbidity.

**Table 3:** Multivariable logistic regression results for non-hydrocodone use.

Non-Hydrocodone Use				
Non-dual-eligible non-Hispanic White				
Policy Change				
	Post policy change	1.29	[1.19, 1.40]	<0.001
	Before policy change (reference)			
Time Trend				
	in 12 months	1.00	[0.98, 1.02]	0.815
Non-dual-eligible racial–ethnic minority				
Policy Change				
	Post policy change	1.22	[0.97, 1.53]	0.087
	Before policy change (reference)			
Time Trend				
	in 12 months	1.03	[0.98, 1.09]	0.178
Dual-eligible				
Policy Change				
	Post policy change	1.12	[0.95, 1.32]	0.178
	Before policy change (reference)			
Time Trend				
	in 12 months	1.01	[0.97, 1.04]	0.727

Note: The regression models were controlled for age groups, income quartiles, percent below poverty level, percent below non-high school degree, radiation, chemotherapy, immunotherapy, hormonal therapy, depression, and Charlson comorbidity.

## Data Availability

The data used in our study are from the SEER-Medicare database, which is managed and provided by the National Cancer Institute (NCI). Information about the database can be found here: https://healthcaredelivery.cancer.gov/seermedicare/ (accessed on 1 January 2025). Access to the SEER-Medicare dataset is available by request following NCI’s data use procedures, detailed here: https://healthcaredelivery.cancer.gov/seermedicare/obtain/requests.html (accessed on 1 January 2025).

## Supplementary Materials

The following supporting information can be downloaded at https://www.mdpi.com/article/10.3390/cancers17132146/s1. Table S1: (a) Sample Description by Hydrocodone Use. (b) Sample Description by Non-hydrocodone Use. Table S2: (a) Multivariable logistic regression results for hydrocodone use—detailed results for cancer treatment and comorbidities. (b) Multivariable logistic regression results for non-hydrocodone use—detailed results for cancer treatment and comorbidities. Table S3: Segmented time series logistic regression results for hydrocodone use and non-hydrocodone use. Table S4: Multivariable logistic regression results for long-term hydrocodone and non-hydrocodone opioid use above 90 days.
