# Supplementary material for: Hydrocodone Rescheduling and Opioid Prescribing Disparities in Breast Cancer Patients
Source: Cancers (Basel). 2025 Jun 25;17(13):2146. doi: 10.3390/cancers17132146 (PMC12248469; doi:10.3390/cancers17132146)
Supplement: Supplementary file 1 [file cancers-17-02146-s001.zip › cancers-3642895-supplementary.pdf]

Supplementary Table S1. (a) Sample Description by Hydrocodone Use; (b) Sample Description by Non-hydrocodone Use.

(a) Sample Description by Hydrocodone Use

	Hydrocodone Use			
	**Yes (N = 23979)**	**No (N = 28327)**	**Total (N = 52306)**	***p*-Value**
**Age**				<.0001[1]
66–69	6417 (26.8%)	6310 (22.3%)	12727 (24.3%)	
70–74	7313 (30.5%)	7964 (28.1%)	15277 (29.2%)	
75–79	5191 (21.6%)	6038 (21.3%)	11229 (21.5%)	
>=80	5058 (21.1%)	8015 (28.3%)	13073 (25.0%)	
**Race/Ethnicity**				0.1951[1]
Non-Hispanic White	19915 (83.1%)	23544 (83.1%)	43459 (83.1%)	
Non-Hispanic Black	1511 (6.3%)	1681 (5.9%)	3192 (6.1%)	
Hispanic/Latino	1363 (5.7%)	1621 (5.7%)	2984 (5.7%)	
Other	1190 (5.0%)	1481 (5.2%)	2671 (5.1%)	
**Income quartile**				<.0001[1]
First quartile	6697 (27.9%)	6715 (23.7%)	13412 (25.6%)	
Second quartile	5753 (24.0%)	7042 (24.9%)	12795 (24.5%)	
Third quartile	5594 (23.3%)	7133 (25.2%)	12727 (24.3%)	
Fourth quartile	5935 (24.8%)	7437 (26.3%)	13372 (25.6%)	
**% below poverty**				<.0001[1]
0%-<5%	5625 (23.5%)	8192 (29.0%)	13817 (26.4%)	
5% to <10%	5286 (22.1%)	6415 (22.7%)	11701 (22.4%)	
10% to <20%	6600 (27.6%)	7275 (25.7%)	13875 (26.6%)	
20% to 100%	6440 (26.9%)	6411 (22.7%)	12851 (24.6%)	
**% of Non-high school degree**				<.0001[1]

(a) Sample Description by Hydrocodone Use

	Hydrocodone Use			
	**Yes (N = 23979)**	**No (N = 28327)**	**Total (N = 52306)**	***p*-Value**
0%-<5%	2514 (10.5%)	3199 (11.3%)	5713 (10.9%)	
5% to <10%	3546 (14.8%)	4396 (15.5%)	7942 (15.2%)	
10% to <20%	5692 (23.8%)	6998 (24.7%)	12690 (24.3%)	
20% to 100%	12199 (50.9%)	13700 (48.4%)	25899 (49.6%)	
**Radiation therapy**				<.0001[1]
Yes	14461 (60.3%)	15994 (56.5%)	30455 (58.2%)	
No	9518 (39.7%)	12333 (43.5%)	21851 (41.8%)	
**Chemotherapy**				<.0001[1]
Yes	7076 (29.5%)	7829 (27.6%)	14905 (28.5%)	
No	16903 (70.5%)	20498 (72.4%)	37401 (71.5%)	
**Immunotherapy**				<.0001[1]
Yes	3529 (14.7%)	3822 (13.5%)	7351 (14.1%)	
No	20450 (85.3%)	24505 (86.5%)	44955 (85.9%)	
**Hormonal therapy**				<.0001[1]
Yes	21776 (90.8%)	25104 (88.6%)	46880 (89.6%)	
No	2203 (9.2%)	3223 (11.4%)	5426 (10.4%)	
**Charlson comorbidity**				0.0297[1]
No	9455 (43.0%)	10803 (41.7%)	20258 (42.3%)	
1	6130 (27.9%)	7301 (28.2%)	13431 (28.1%)	
2	3675 (16.7%)	4435 (17.1%)	8110 (16.9%)	
3 or more	2716 (12.4%)	3338 (12.9%)	6054 (12.7%)	
**Policy change**				<.0001[1]

(a) Sample Description by Hydrocodone Use

	Hydrocodone Use			
	**Yes (N = 23979)**	**No (N = 28327)**	**Total (N = 52306)**	***p*-Value**
Pre policy	10887 (45.4%)	9367 (33.1%)	20254 (38.7%)	
Post policy	13092 (54.6%)	18960 (66.9%)	32052 (61.3%)	

(b) Sample Description by Non-hydrocodone Use

	Non-hydrocodone Opioids			
	**Yes (N = 24897)**	**No (N = 27409)**	**Total (N = 52306)**	***p*-Value**
**Age**				<.0001[1]
66–69	6503 (26.1%)	6224 (22.7%)	12727 (24.3%)	
70–74	7613 (30.6%)	7664 (28.0%)	15277 (29.2%)	
75–79	5329 (21.4%)	5900 (21.5%)	11229 (21.5%)	
>=80	5452 (21.9%)	7621 (27.8%)	13073 (25.0%)	
**Race/Ethnicity**				<.0001[1]
Non-Hispanic White	20571 (82.6%)	22888 (83.5%)	43459 (83.1%)	
Non-Hispanic Black	1708 (6.9%)	1484 (5.4%)	3192 (6.1%)	
Hispanic/Latino	1514 (6.1%)	1470 (5.4%)	2984 (5.7%)	
Other	1104 (4.4%)	1567 (5.7%)	2671 (5.1%)	
**Income quartile**				0.0083[1]
First quartile	6216 (25.0%)	7196 (26.3%)	13412 (25.6%)	
Second quartile	6173 (24.8%)	6622 (24.2%)	12795 (24.5%)	
Third quartile	6091 (24.5%)	6636 (24.2%)	12727 (24.3%)	
Fourth quartile	6417 (25.8%)	6955 (25.4%)	13372 (25.6%)	
**% below poverty**				0.0019[1]
0%-<5%	6720 (27.0%)	7097 (25.9%)	13817 (26.4%)	

(b) Sample Description by Non-hydrocodone Use

	Non-hydrocodone Opioids			
	Yes (N = 24897)	No (N = 27409)	Total (N = 52306)	*p*-Value
5% to <10%	5572 (22.4%)	6129 (22.4%)	11701 (22.4%)	
10% to <20%	6630 (26.7%)	7245 (26.5%)	13875 (26.6%)	
20% to 100%	5949 (23.9%)	6902 (25.2%)	12851 (24.6%)	
**% of Non-high school degree**				0.2443[1]
0%-<5%	2674 (10.8%)	3039 (11.1%)	5713 (10.9%)	
5% to <10%	3818 (15.4%)	4124 (15.1%)	7942 (15.2%)	
10% to <20%	6109 (24.6%)	6581 (24.0%)	12690 (24.3%)	
20% to 100%	12270 (49.3%)	13629 (49.8%)	25899 (49.6%)	
**Radiation therapy**				<.0001[1]
Yes	14766 (59.3%)	15689 (57.2%)	30455 (58.2%)	
No	10131 (40.7%)	11720 (42.8%)	21851 (41.8%)	
**Chemotherapy**				<.0001[1]
Yes	7819 (31.4%)	7086 (25.9%)	14905 (28.5%)	
No	17078 (68.6%)	20323 (74.1%)	37401 (71.5%)	
**Immunotherapy**				<.0001[1]
Yes	3680 (14.8%)	3671 (13.4%)	7351 (14.1%)	
No	21217 (85.2%)	23738 (86.6%)	44955 (85.9%)	
**Hormonal therapy**				<.0001[1]
Yes	22668 (91.0%)	24212 (88.3%)	46880 (89.6%)	
No	2229 (9.0%)	3197 (11.7%)	5426 (10.4%)	
**Charlson comorbidity**				<.0001[1]
No	9095 (40.1%)	11163 (44.3%)	20258 (42.3%)	

(b) Sample Description by Non-hydrocodone Use

	Non-hydrocodone Opioids			
	**Yes (N = 24897)**	**No (N = 27409)**	**Total (N = 52306)**	***p*-Value**
1	6366 (28.1%)	7065 (28.1%)	13431 (28.1%)	
2	4033 (17.8%)	4077 (16.2%)	8110 (16.9%)	
3 or more	3177 (14.0%)	2877 (11.4%)	6054 (12.7%)	
**Policy change**				<.0001[1]
Pre policy	8785 (35.3%)	11469 (41.8%)	20254 (38.7%)	
Post policy	16112 (64.7%)	15940 (58.2%)	32052 (61.3%)	

[1] Chi-Square *p*-value.

Supplementary Table S2. (a) Multivariable logistic regression results for hydrocodone use – detailed results for cancer treatment and comorbidities. (b) Multivariable logistic regression results for non-hydrocodone use – detailed results for cancer treatment and comorbidities.

(a)										
		**Dual-eligible non-Hispanic White**			**Dual-eligible racial-ethnic minority**			**Non-dual-eligible**		
**Variables**		**AOR**	**95% CI**	***p*-value**	**AOR**	**95% CI**	***p*-value**	**AOR**	**95% CI**	***p*-value**
Radiation										
	Yes	1.16	[1.03 , 1.31]	0.0141	1.18	[1.03 , 1.34]	0.0151	1.08	[1.04 , 1.13]	0.0003
	Ref = No									
Chemotherapy										
	Yes	1.14	[0.98 , 1.32]	0.0888	1.22	[1.04 , 1.43]	0.0162	1.12	[1.07 , 1.17]	0.0000
	Ref = No									
Immunotherapy										
	Yes	1.06	[0.88 , 1.28]	0.5319	0.95	[0.78 , 1.17]	0.6445	1.10	[1.04 , 1.17]	0.0012
	Ref = No									
Hormonal therapy										
	Yes	1.06	[0.90 , 1.24]	0.4879	1.35	[1.11 , 1.63]	0.0023	1.26	[1.18 , 1.35]	0.0000
	Ref = No									
Charlson Comorbidity										
	1	1.16	[1.00 , 1.35]	0.0485	0.99	[0.83 , 1.18]	0.8962	0.95	[0.90 , 0.99]	0.0176
	2	1.10	[0.94 , 1.29]	0.2481	1.01	[0.85 , 1.21]	0.8837	0.95	[0.89 , 1.00]	0.0683
	3	0.98	[0.84 , 1.14]	0.7859	1.01	[0.86 , 1.19]	0.8917	0.96	[0.89 , 1.03]	0.2380
	Ref = No									

(b)										
		**Non-dual-eligible non-Hispanic White**			**Non-dual-eligible racial-ethnic minority**			**Dual-eligible**		
**Variables**		**AOR**	**95% CI**	***p*-value**	**AOR**	**95% CI**	***p*-value**	**AOR**	**95% CI**	***p*-value**
Radiation										
	Yes	0.97	[0.93 , 1.02]	0.248	0.99	[0.88 , 1.12]	0.914	1.07	[0.98 , 1.17]	0.140
	Ref = No									
Chemotherapy										
	Yes	1.17	[1.12 , 1.23]	<0.001	1.32	[1.15 , 1.51]	<0.001	1.29	[1.16 , 1.44]	<0.001
	Ref = No									
Immunotherapy										
	Yes	1.01	[0.95 , 1.08]	0.664	0.99	[0.84 , 1.18]	0.927	0.87	[0.76 , 1.00]	0.042
	Ref = No									
Hormonal therapy										
	Yes	1.20	[1.12 , 1.29]	<0.001	1.07	[0.88 , 1.31]	0.496	1.22	[1.08 , 1.38]	0.001
	Ref = No									
Charlson Comorbidity										
	1	1.09	[1.04 , 1.15]	<0.001	1.05	[0.91 , 1.21]	0.487	0.99	[0.88 , 1.11]	0.848
	2	1.21	[1.14 , 1.29]	<0.001	1.30	[1.10 , 1.53]	0.002	1.08	[0.96 , 1.21]	0.219
	3	1.33	[1.23 , 1.44]	<0.001	1.30	[1.08 , 1.57]	0.006	1.28	[1.15 , 1.43]	<0.001
	Ref = No									

Supplementary Table S3. Segmented time series logistic regression results for hydrocodone use and non-hydrocodone use

Hydrocodone Use

	AOR	95% CI	*p*-value
Dual-eligible non-Hispanic White			
Time trend before policy change (per year)	0.87	[0.8, 0.93]	<0.001
Immediate Policy Change	0.77	[0.61, 0.97]	0.03
Post-policy slope change (per year)	1.07	[0.96, 1.19]	0.22
Dual-eligible racial-ethnic minority			
Time trend before policy (per year)	0.91	[0.84, 0.99]	0.03
Immediate Policy Change	0.62	[0.47, 0.82]	<0.001
Post-policy slope change (per year)	1.04	[0.92, 1.17]	0.54
Non-dual-eligible			
Time trend before policy change (per year)	0.90	[0.87, 0.94]	<0.001
Immediate Policy Change	0.84	[0.77, 0.92]	<0.001
Post-policy slope change (per year)	1.01	[0.97, 1.05]	0.62

Non-Hydrocodone Use

	AOR	95% CI	*p*-value
Non-dual-eligible non-Hispanic White			
Time trend before policy change (per year)	1.07	[1.04, 1.09]	<0.001
Immediate Policy Change	1.23	[1.12, 1.34]	<0.001
Post-policy slope change (per year)	0.91	[0.87, 0.94]	<0.001
Non-dual-eligible racial-ethnic minority			
Time trend before policy change (per year)	1.01	[0.90, 1.13]	0.88
Immediate Policy Change	1.24	[0.97, 1.59]	0.08
Post-policy slope change (per year)	1.04	[0.91, 1.18]	0.61
Dual-eligible			
Time trend before policy change (per year)	1.06	[1.02, 1.11]	0.01
Immediate Policy Change	1.09	[0.93, 1.28]	0.30
Post-policy slope change (per year)	0.91	[0.86, 0.97]	<0.001

Supplementary Table S4. Multivariable logistic regression results for long-term hydrocodone and non-hydrocodone opioid use above 90 days

Hydrocodone Use

Variables	AOR	95% CI	*p*-value
Dual-eligible non-Hispanic White			
Policy Change			
Post policy change	0.81	[0.58 , 1.14]	0.228
Before policy change (reference)			
Time Trend			
in 12 months	0.98	[0.91 , 1.05]	0.574
Dual-eligible racial-ethnic minority			
Policy Change			
Post policy change	0.88	[0.54 , 1.44]	0.609
Before policy change (reference)			
Time Trend			
in 12 months	0.94	[0.84 , 1.04]	0.231
Non-dual-eligible			
Policy Change			
Post policy change	1.03	[0.83 , 1.29]	0.783
Before policy change (reference)			
Time Trend			
in 12 months	0.89	[0.85 , 0.93]	<0.001

Non-Hydrocodone Use

	AOR	95%CI	*p*-value
Non-dual-eligible non-Hispanic White			
Policy Change			
Post policy change	0.65	[0.42 , 1.01]	0.056
Before policy change (reference)			
Time Trend			
in 12 months	0.99	[0.90 , 1.09]	0.902
Non-dual-eligible racial-ethnic minority			
Policy Change			
Post policy change	0.96	[0.37 , 2.51]	0.931
Before policy change (reference)			
Time Trend			
in 12 months	0.91	[0.74 , 1.12]	0.390
Dual-eligible			
Policy Change			
Post policy change	0.84	[0.46 , 1.54]	0.576
Before policy change (reference)			
Time Trend			
in 12 months	0.98	[0.86 , 1.12]	0.783
